# Obstructed Hemivagina and Ipsilateral Renal Agenesis Syndrome in a Neonate

**DOI:** 10.7759/cureus.36822

**Published:** 2023-03-28

**Authors:** Luna Khanal, Kanika Deora, Suman R Gaihre, Bajaj Monika

**Affiliations:** 1 Pediatrics, Children's Hospital of Michigan, Detroit, USA; 2 Neonatology, Central Michigan University College of Medicine, Mount Pleasant, USA

**Keywords:** uterine didelphys, renal dysplasia, management, newborn, ohvira syndrome

## Abstract

Obstructed hemivagina with an ipsilateral renal anomaly (OHVIRA) syndrome is a congenital malformation that presents as a uterine didelphys with an obstructed hemivagina and an associated ipsilateral renal aberration. The clinical symptoms usually manifest after menarche. Unlike the typical presentation in adolescence, this case report features a neonatal presentation of OHVIRA syndrome with an unusual renal association. A female twin delivered at 35 weeks of gestation was transferred to our institution after birth from an outside hospital due to respiratory distress and for evaluation of the left multicystic dysplastic kidney identified on prenatal ultrasound. Physical examination and lab results, including a complete blood count, and a basic metabolic panel, including blood urea and serum creatinine, were within the normal range for age. Abdominal and pelvic ultrasound showed multicystic dysplastic left pelvic kidney, congenital hepatic cyst measuring 6 mm, uterine didelphys with duplication of the vaginal canal, and obstructed left hemivagina corresponding to the OHVIRA syndrome. Further testing revealed a normal chromosomal microarray, small patent foramen ovale on the echocardiogram, no vertebral or rib anomalies on the spinal x-ray, normal hearing test, and mild optic cupping on the ophthalmological evaluation. The pediatric surgeon and urologist recommended an outpatient follow-up and elective surgery in the future. This is a unique case presenting in the neonatal period with an unusual association. Timely intervention can help prevent obstetric complications.

## Introduction

Obstructed hemivagina with the ipsilateral renal anomaly (OHVIRA) syndrome, previously known as Herlyn-Werner-Wunderlich syndrome, is a congenital malformation characterized by uterus didelphys, obstructed hemivagina, and an ipsilateral renal abnormality, as a result of abnormal development of the paramesonephric and mesonephric duct [1,2]. Its incidence ranges from 0.1 to 3.8% in females [3]. This syndrome has been classified as complete or incomplete (based on the characteristics of the vaginal septum) and the possible communication between the duplicated cervixes [4,5]. Clinical manifestations mainly occur during puberty following menarche, which includes progressive dysmenorrhea, nonspecific lower abdominal pain, persistent vaginal discharge, urinary retention, and pelvic mass. There is no reported relation to certain ethnicities or family history for this syndrome. While renal agenesis is the classic renal presentation, other associated renal anomalies include renal dysplasia and ectopic ureters. Renal malformations occur mostly ipsilateral to the obstructed hemivagina. However, 50% of cases of contralateral renal anomalies have been documented. The diagnosis requires a high index of suspicion, and efforts should be made to identify uterovaginal abnormalities when encountering patients with dysplastic or missing kidneys [6]. Due to routine prenatal ultrasonographs, this malfunction is often identified during the evaluation of a dysplastic, atrophic, or absent kidney [2]. Ultrasound scans and magnetic resonance imaging are the keys to diagnosis and management plans. Early management helps to prevent complications like pelvic inflammatory disease, endometriosis, pyometra, and infertility [7]. Despite several publications on adolescents with this syndrome, there is limited evidence of diagnosis and management of such patients during the neonatal period. Therefore, our case emphasizes the unusual presentation in a newborn, which makes it noteworthy.

This case was previously presented at a poster presentation at the 2022 Michigan American Academy of Pediatrics (MIAAP) annual conference on September 30, 2022.

## Case presentation

A female twin, born at 35 weeks of gestation via C-section due to maternal pre-eclampsia to a 37 year G2 P2 mother was referred to our neonatal intensive care unit (NICU) from an outside hospital on day one of life due to respiratory distress following birth and for the evaluation of the left multicystic dysplastic kidney that was identified during the prenatal ultrasound. The infant required positive pressure ventilation after birth and was transferred to a high-flow nasal cannula due to persistent tachypnea and respiratory distress. Physical examination on admission revealed no dysmorphism, mild tachypnea and retractions, normal heart sounds, sacral dimple with a visible base, and normal ano-genitalia for age. The baby continued to make adequate urine and passed meconium after birth; she was gradually weaned to room air in the first week of life.

**Figure 1:**
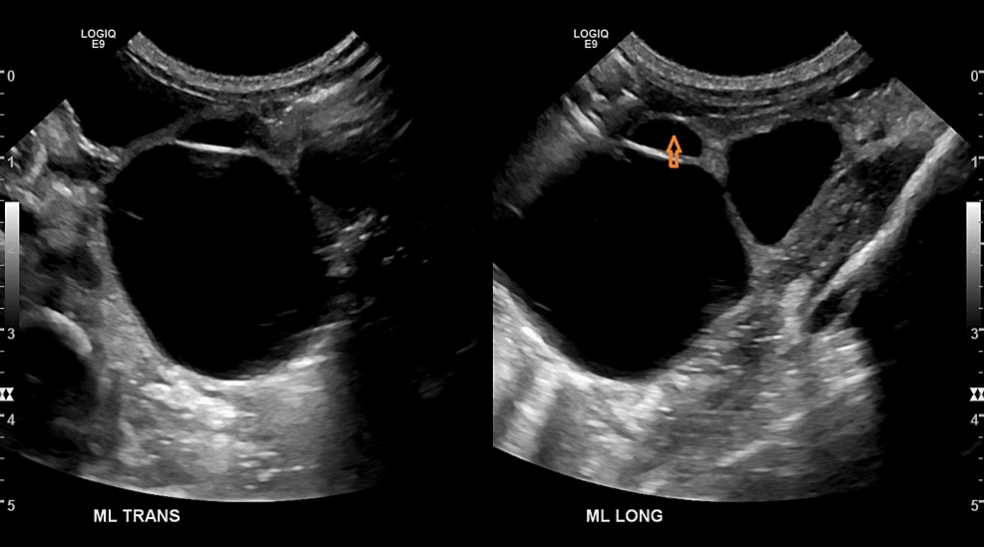
The arrow in the picture points to the multicystic dysplastic kidney that was identified in the ultrasound.

**Figure 2:**
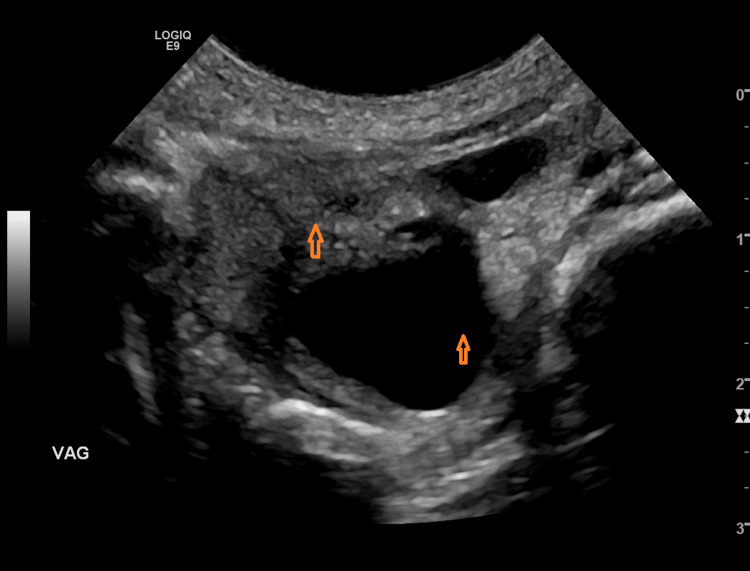
The arrow points to the uterine didelphys, showing two endometrial cavities, and the arrow on the downward portion points to the obstructed hemivagina.

Complete blood count, electrolytes, urea, and serum creatinine obtained at admission were within the normal range for age. Abdominal ultrasound confirmed the presence of a multicystic dysplastic left pelvic kidney (Figure 1), the normal appearance of the right kidney, an anechoic thin-walled 6 mm structure in the right hepatic lobe corresponding to a congenital hepatic cyst, and didelphys configuration of the uterus with duplication of the vaginal canal with anechoic fluid suspicious for unilateral obstructed hemivagina (Figure 2), corresponding to the OHVIRA syndrome.

Pediatrics, genetics, surgery, and urology were consulted. Chromosomal microarray, echocardiogram, and spinal x-ray were obtained as part of the work-up, and all the tests were reported to be normal. The baby had normal hearing, and mild optic cupping was noted on an ophthalmological exam. The infant remained stable with no signs of bladder outlet obstruction. No surgical intervention was performed during this admission. Urology and pediatric surgery were arranged for an outpatient follow-up with a plan to schedule elective surgery. A pre-operative MRI would be obtained to delineate the anatomy.

The patient remained in the NICU for three weeks for prematurity and was later transferred to the pediatrics floor. She was discharged two days later with no concerns.

## Discussion

OHVIRA syndrome is mostly diagnosed following menarche. Symptoms include hematocolpos, pelvic pain, vaginal or pelvic mass, abnormal vaginal discharge, abdominal pain, vomiting, fever, and acute urinary retention. Recent literature suggests that the age at diagnosis has dropped significantly due to increased awareness and availability of prenatal ultrasounds. In a retrospective analysis involving 43 prepubertal patients, the average age of diagnosis was 1.3 months. It required follow-up for at least 25.5 months, among which 14% required surgery around 31.2 months due to various complications [2].

Approximately 37-60% of females diagnosed with congenital unilateral renal agenesis have genital anomalies [8]. Ipsilateral renal agenesis is the most common urologic anomaly. At the same time, other reported malformations include renal duplication, multicystic dysplastic kidney, vesicoureteric reflux of the contralateral urinary tract, and ectopic ureteric orifice to the obstructed vagina. Our patient also presented with a unilateral multicystic dysplastic kidney [1,3,8]. Uterine didelphys is the most common uterine anomaly reported in our case. A case series including 42 patients reported a 22% incidence of a septate uterus. In contrast to uterine didelphys, septate uteri are commonly associated with infertility and obstetric complications during pregnancy, so they need hysteroscopic metroplasty [9]. Another case series and systematic review of 724 cases showed the most common variant was left obstructed hemivagina in 50.7% of patients, with isolated hematocolpos or hydrocolpos seen in 55.9%, uterus didelphys in 82.9%, and ipsilateral renal agenesis in 92.2%; 7.8% of cases of dysplastic or multicystic kidney were reported. The most common surgical intervention was vaginal septectomy (86.5%); other interventions included hemivaginectomy, hemihysterectomy, total hysterectomy, salpingectomy, or oophorectomy. About 7.5% of infants had spontaneous resolution of hydrocolpos [3,8].

Diagnosis of OHVIRA syndrome is suspected on renal and pelvic ultrasound, and MRI is the gold standard. It should be done prior to menarche and helps to define the anatomy before surgical correction [1]. Most infants show spontaneous resolution of hydrocolpos in the first few months and can be managed conservatively, as in our case. Drainage or early vaginal septectomy should be performed in cases of recurrent urinary tract infections (UTIs), urinary incontinence, abdominal pain, or pyocolpos causing a non-resolving large vaginal mass. Nephroureterectomy is indicated in the case of the non-functioning multicystic dysplastic kidney with an ectopic ureteric orifice [3,8]. Following surgical intervention, regular interval ultrasounds every six months for the first two years, followed by annual and biennial ultrasounds, should be performed to monitor for the recurrence of obstructed hemivagina, which could commonly occur, and to monitor for any renal complications secondary to renal aberration.

In our case, the patient was stable with no signs of bladder outlet obstruction; hence, no intervention was performed in the neonatal period. It was planned to follow her up as an outpatient with routine interval ultrasound and monitoring of kidney function. Patients require regular monitoring of renal function as there is a risk of deterioration of renal function, hypertensive disease in pregnancy, proteinuria, and chronic renal and cardiovascular disease secondary to the multicystic dysplastic kidney [2,3,6,8].

## Conclusions

Our case underscores the importance of evaluating females with renal anomalies for obstructed hemivagina and ipsilateral renal anomaly syndrome. Even though the typical manifestations of this syndrome are seen during pubertal age, the increased popularity of prenatal ultrasound has led to early identification of this syndrome, as in our case. Most of the patients can be managed conservatively with close follow-up. Timely diagnosis and intervention may reduce these patients' risk of obstetric and renal complications. Hence, it is important to have knowledge of this unique anomaly, especially among pediatricians and radiologists. 
